# Temozolomide resistance in glioblastoma occurs by miRNA-9-targeted PTCH1, independent of sonic hedgehog level

**DOI:** 10.18632/oncotarget.2778

**Published:** 2015-02-06

**Authors:** Jessian L. Munoz, Vivian Rodriguez-Cruz, Shakti H. Ramkissoon, Keith L. Ligon, Steven J. Greco, Pranela Rameshwar

**Affiliations:** ^1^ New Jersey Medical School, Rutgers, Newark, NJ, USA; ^2^ Graduate School of Biomedical Science, Rutgers School of Biomedical Health Sciences, Newark, NJ, USA; ^3^ University of Puerto Rico, Chemistry Department, Cayey, Puerto Rico; ^4^ Department of Pathology, Brigham and Women’s Hospital, Boston Children’s Hospital and Harvard Medical School, Department of Medical Oncology, Dana-Farber Cancer Institute, Boston, MA, USA

**Keywords:** glioblastoma, MicroRNA-9, PTCH, Temozolomide, P-gp

## Abstract

Glioblastoma Multiforme (GBM), the most common and lethal adult primary tumor of the brain, showed a link between Sonic Hedgehog (SHH) pathway in the resistance to temozolomide (TMZ). PTCH1, the SHH receptor, can tonically represses signaling by endocytosis. We asked how the decrease in PTCH1 in GBM cells could lead to TMZ-resistance. TMZ resistant GBM cells have increased PTCH1 mRNA and reduced protein. Knockdown of Dicer, a Type III RNAase, indicated that miRNAs can explain the decreased PTCH1 in TMZ resistant cells. Computational studies, real-time PCR, reporter gene studies, western blots, target protector oligos and ectopic expression identified miR-9 as the target of PTCH1 in resistant GBM cells with concomitant activation of SHH signaling. MiR-9 mediated increases in the drug efflux transporters, MDR1 and ABCG2. MiR-9 was increased in the tissues from GBM patients and in an early passage GBM cell line from a patient with recurrent GBM but not from a naïve patient. Pharmacological inhibition of SHH signaling sensitized the GBM cells to TMZ. Taken together, miR-9 targets PTCH1 in GBM cells by a SHH-independent method in GBM cells for TMZ resistance. The identified pathways could lead to new strategies to target GBM with combinations of drugs.

## INTRODUCTION

Gliobastoma (GBM), the most common and aggressive adult human primary brain tumor with a median survival of 11 months and 5% survival at 5 years of diagnosis. The treatment for GBM includes surgery, radiotherapy and, Temozolomide (TMZ) or Bevacizumab (Avastin^®^) [1]. In a recent report, Bevacizumab, which is a humanized monoclonal antibody to vascular endothelial growth factor A, has been reported to have little improvement as compared to the standard treatment and, with a worse quality of life for the patients [2].

The cellular pathways involved in the growth and survival of GBM include phosphoinositol-3-phosphate Kinase (PI3K), mitogen activated protein kinase (MAPK), and sonic hedgehog (SHH) [3, 4]. SHH is a dual lipidated 25 kDa secreted mitogen with paracrine and autocrine function in development [5]. A 45 kDa SHH pre-propeptide is autocatalyzed by the C-terminus, resulting in an active N-terminus fragment, SHH-N [6]. A palmitate is added to the N-terminus of SHH-N, which interacts with the transporter, Dispatched, for secretion [7]. The interaction between SHH and its 12-transmembrane Patched (PTCH1) receptor de-represses SMO, which signals through a G-protein receptor complex [8]. SHH signaling mainly occurs by the transcriptional factor, glioma-associated oncogene 1 (Gli1), which is a downstream of SMO. The over-activation of SHH signaling has been implicated in several tumors, including GBM [9].

MicroRNAs (miRs) are small, non-coding RNAs that are involved in biological processes such as neural patterning, development and oncogenesis [10, 11]. MiRs are considered as post-transcriptional regulators of genes [12]. This occurs through their interactions with the 3′ UTR of transcripts (mRNA) to suppress translation. MiRs and their targets are generally conserved, suggesting their critical role in development [13].

MiR can regulate the functions of SHH by targeting SMO and Gli1 [14]. MiR-9 has been implicated in genes linked to neurodevelopment such as those associated with chromatin remodeling and malignancy [15, 16]. There are three functional loci for miR in the human genome, miR-9-1, -2 and -3 [17]. We previously reported on an increase in miR-9 in TMZ-resistant GBM cells. We also showed that this miRNA could be targeted by delivering its anti-miRNA in stem cells [18]. MiR-9 regulates key genes in neurodevelopment by synergising with its complementary miR-9*. In this relationship, miR-9 targets REST and miR-9* targets Co-REST [14, 19].

Since mesenchymal stem cells and neural stem cells can be used in therapeutic delivery for GBM, there is a need to further study the mechanism by which miR-9 is involved in GBM resistance [20–22]. This study reports on the molecular mechanisms by which TMZ-induced miR-9 in GBM cells. We showed that miR-9 suppresses PTCH1, which sustained signaling. We also showed that this signaling occurred in a ligand-independent manner.

## RESULTS

### SHH-independent resistance to TMZ

**Figure 1:**
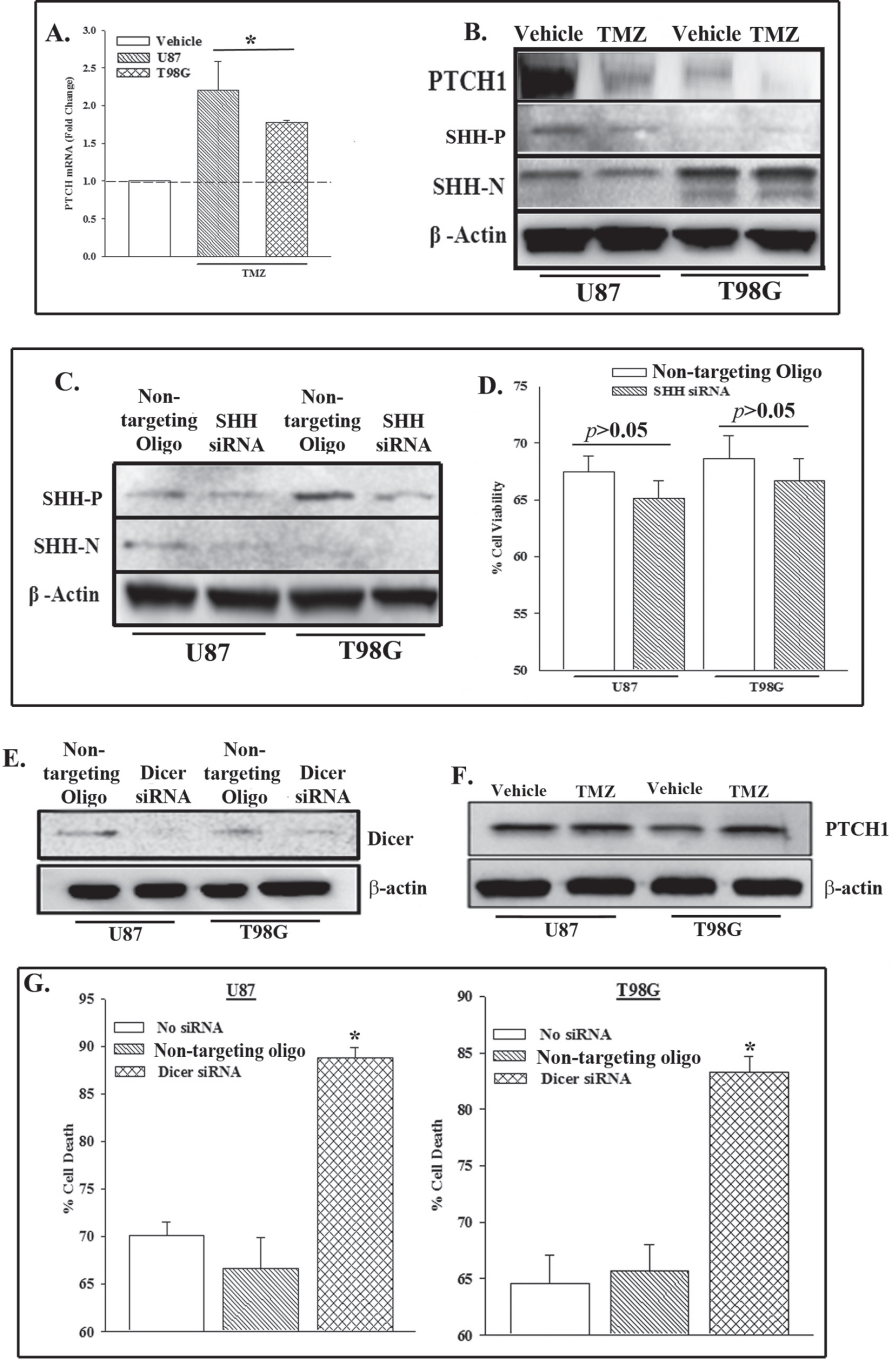
PTCH1 repression in TMZ-treated GBM cells involved miRNA U87 and T98G cells were treated with 200 μM TMZ. At 72 h, real time PCR was performed for PTCH1 mRNA **(A)** and western blot for PTCH1 and SHH (precursor (P) and N-terminus (N)) and β-actin. The values for the untreated group were assigned 1 and are represented by an open bar. The changes in the treated cells were presented as fold change over the vehicle-treated cells. **(B)** U87 and T98G were knocked down for SHH with siRNA and then analyzed for SHH (precursor, P and mature, N) and β-actin by western blot. **(C)** The knocked down cells in ‘C’ were treated with TMZ. At 72 h, the cells were assessed for viability with Cell Titer Blue. The data are presented as the mean % viability relative to vehicle treated siRNA transfected cells ±SD, *n* = 4. **(D)** U87 and T98G were knocked down for *Dicer1*. Control was transfected with non-targeting oligo. Western blot was performed for Dicer. The membrane was stripped and reprobed for β-actin. **(E)** The cells in ‘E’ were treated with 200 μM TMZ. At 72 h, western blot was performed for PTCH1. The membrane was stripped and reprobed for β-actin **(F)** and assessed for viability. **(G)**   *p* < 0.05 vs. control and non-targeting oligo.

SHH signaling has been proposed as a target of anti-neoplastic agents, such as TMZ [23, 24]. SHH signaling occurs with decreased PTCH1 [25]. This study investigated if TMZ resistant cells showed a decrease in PTCH1. GBM cells treated for 72 h resulted in ~30% viability. The surviving cells expressed high levels of the MDR1 gene and resisted TMZ [26]. We established TMZ-resistant U87 and T98G cells and then studied them for PTCH mRNA and protein by qPCR and western blot, respectively. Figure 1A show the fold change in PTCH mRNA. The vehicle-treated point for each cell line was normalized to 1. Since a similar normalization was performed for each cell line, one bar is shown to represent both cell lines. The changes in TMZ-treated cells were presented as fold change over the vehicle-treatment. PTCH mRNA was significantly (*p* < 0.05) increased in the TMZ resistant cells (Figure 1A) whereas its corresponding protein was decreased (Figures 1B, S1). These results suggested that TMZ could induce post-transcriptional regulation of *PTCH1.*

The interaction between PTCH1 and its ligand, SHH, can lead to endocytic degradation of PTCH [27]. Whole cell extracts from TMZ- and vehicle- treated U87 and T98G were studied for intracellular SHH by western blot. TMZ did not cause any change in the band densities for the precursor (SHH-P) or the mature ligand (SHH-N) (Figures 1B, S1). Due to the sustained level of intracellular SHH, we asked if this could be explained by its role in protecting the GBM cells from TMZ treatment. We compared SHH knockdown GBM cells with cells transfected with a non-targeting oligo. The efficiency of SHH knockdown is shown in Figures 1C, S2. The presence or absence of SHH did not change the pattern of TMZ resistance since there was no significant (*p* > 0.05) change in cell viability between the control and knockdown cells (Figure 1D). These results indicated that SHH was not involved in the resistance of GBM to TMZ.

### Dicer-knockdown sensitized GBM cells to TMZ

The level of SHH in the untreated and TMZ-treated GBM cells could not explain why PTCH1 protein was decreased in TMZ resistant GBM cells (Figure 1B). Although PTCH protein was decreased its corresponding mRNA was increased in the TMZ-treated GBM cells (Figures 1A, 1B). This indicated that the translation of PTCH1 mRNA was suppressed. We asked if miRNA could be responsible for the suppressed translation [10]. We blocked the processing of all miRNAs by knockdown of RNase type III Dicer in U87 and T98G cells and then compared with non-targeting oligo transfectants (control). Western blot with whole cell extracts verified dicer knockdown (Figures 1E, S3). Western blots with whole cells extracts from TMZ-treated dicer knockdown cells failed to decrease PTCH1 protein (Figures 1F, S4). Together, these studies supported a role for miRNA in the suppression of PTCH1 translation in TMZ-resistant GBM cells.

Since dicer knockdown maintained high expression of PTCH1 protein (Figure 1F), we asked if the sustained presence of PTCH1 sensitized the GBM cells to TMZ. U87 and T98G cells were knockdown for dicer or transfected with non-targeting oligos and then treated with 200 μM TMZ. After 72 h, we performed viability studies. The results, presented as % cell death, showed a significant (*p* < 0.05) increase in cell death when dicer was knocked down as compared to non-targeting oligo control (Figure 1G). Considering the relevance of dicer in miRNA processing [28], the results indicated that miRNAs suppress PTCH1 and this induced TMZ resistance.

### MiR-9 in the resistance of GBM to TMZ

**Figure 2:**
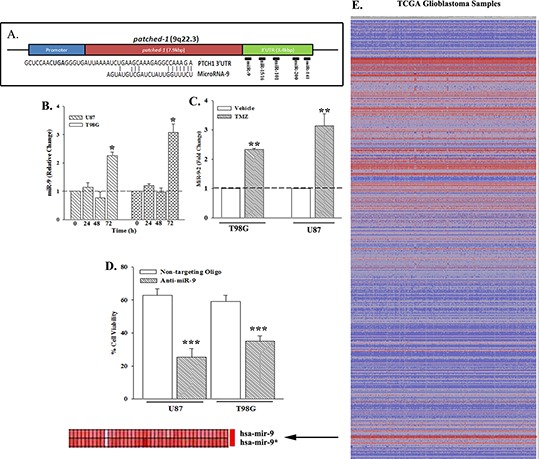
Increased miR-9 in GBM **(A)** Computational analyses were performed for potential miRNA interacting site with the PTCH1 3′ UTR. The alignment between the sequence for miR-9 and the 3′ UTR of *PTCH1* is shown below. **(B)** U87 and T98G cells were treated with 200 μM TMZ. At various times, real time PCR (Taqman) was performed for miR-9. **(C)** The studies in ‘C’ were repeated, except for studies at the 72-h time point, and with primers for miR-9-2, using Sybr-Green. **(D)** U87 and T98G were transfected with non-targeting oligo or anti-miR-9 and then treated with 200 μM TMZ. After 72 h, cell viability was assessed and presented as % viability, *n* = 4 ±SD. **(E)** The heat map shows the analyses from 505 miRNA arrays with GBM tissues from The Cancer Genome Atlas (TCGA). The map represents the miRNAs with +/– 0.5-fold changes from the internal control. Arrow highlights the output for miR-9. *p < 0.05 vs. other time points, **p < 0.05 vs. vehicle. ***p < 0.05 vs. non-targeting oligo.

This set of studied sought the identity of the candidate miRNA(s) in TMZ resistance. We focused on the miRNA(s) that could target PTCH1. An analysis of the 3′ UTR of *PTCH1* identified potential interacting sites for mir-9, mir-16, mir-101, mir-141 and mir-200a (Figure 2A, Table S1). Time-course real time PCR with viable TMZ-treated GBM cells indicated a significant (*p* < 0.05) increase in miR-9 at 72 h (Figure 2B).

Mature miR-9 can be derived from three different loci at distinct chromosomes: miR-9-1, -2 and/or -3. Real-time PCR with RNA from TMZ-treated GBM cells with primers specific for the three different miRs [29] indicated a significant (*p* < 0.05) increase in miR-9-2 (Figure 2C). We next asked if miR-9 is responsible for TMZ resistance by studying the viability of miR-9-knockdown GBM cells that were treated with 200 μM of TMZ for 72 h. Control studies used GBM cells transfected with non-targeting oligo. The knockdown cells (anti-miR-9) showed a significant (*p* < 0.05) decrease in cell viability as compared to control (Figure 2D). This finding was significant considering that miR-9 has been linked to neurogenesis and malignancy [15, 30].

We analyzed the Cancer Genome Atlas (TCGA) with >500 different GBM samples for miR-9 expression (Figure 2E). There was >2 fold increase in miR-9, shown in the enlarged section of the heat map (Figure 2E, lower left, arrow). Together, this section indicated that miR-9 is increased in TMZ resistant GBM cells and primary GBM cells.

### MiR-9 targets PTCH1 mRNA

TMZ-resistant GBM cells showed an increase in miR-9 and a decrease in PTCH1 (Figures 1, 2). We therefore investigated if miR-9 can target PTCH1. This question was addressed with a reporter gene system (luciferase) and CCL64 cells. This cell line was a good model to study the relationship between miR-9 and *PTCH1* expression because they do not express *miR-9* but expressed *PTCH1* (Figure S5).

**Figure 3:**
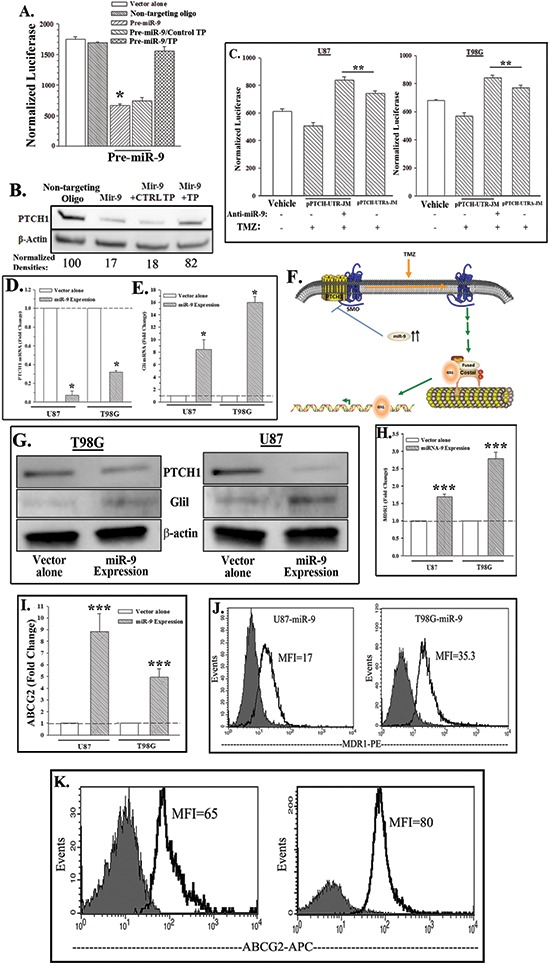
MiR-9 targets PTCH1 and activates SHH signaling and drug transporters **(A & B)** CCL64, stably transfected with pPTCH-UTR-JM, was transiently transfected with pre-miR-9, non-targeting oligo and/or target protection (TP) oligo. After 48 h, luciferase activities were quantitated and then presented as normalized luciferase ±SD, *n* = 4; TP: Target Protector (A). Western blots were performed with whole cell extracts for PTCH1 and β-actin. The normalized band densities are shown at the bottom of the images (B). **(C)** U87 and T98G, stably transfected with pPTCH-UTR-JM or pPTCH-UTR™-JM, were transiently transfected with anti-miR-9 and then treated with 200 μM TMZ or vehicle. After 48 h, luciferase activities were quantitated and the results are presented as mean RLU ±SD, *n* = 4. **(D & E)** MiR-9 was ectopically expressed in U87 and T98G cells. Total RNA was isolated and then studied by real time PCR for PTCH1 mRNA (D) or Gli1 (E). **(F)** Diagram show increased miR-9 in TMZ-treated GBM cells leading to activated SHH signaling. **(G)** Western blots with whole cell extracts were performed for Gli1, PTCH1 and β-actin using miR-9-transfected or vector-transfected GBM cells. **(H–K)** U87 and T98G cells were ectopically expressed for miR-9 or transfected with vector alone. Real-time PCR was performed for MDR1 (H) and ABCG2 (I). The values for control vector in the real time were normalized to 1 for fold-change of the miR-9 transfectants, mean±SD, *n* = 4; flow cytometry for membrane MDR1 (J) and ABCG2 (K). *p < 0.05 vs. Pre-mir-9 and TP, **p < 0.05 vs. pPTCH-UTR-JM. ***p < 0.05 vs. vector alone.

CCL64 cells were stably transfected with a reporter gene vector in which the 3′UTR was inserted downstream of luciferase (pPTCH-UTR-JM). Parallel cultures were transfected with vector alone. The stable transfectants were transiently transfected with pre-miR-9 or a non-targeting oligo (control). Pre-miR-9 significantly (*p* < 0.05) decreased luciferase as compared to the control (Figure 3A). The specificity of the pre-miR-9 was studied with a target protection oligo (TP). The TP competed with pre-miR-9 for the interacting site on the 3′ UTR of PTCH1. The TP prevented miR-9 to decrease luciferase levels (Figure 3A, hatched bar). Western blots for PTCH1 with whole cell extracts from the reporter gene transfectants indicated a decrease in PTCH1 protein by transfected anti-miR-9 (Figure 3B). This was specific since the effect was reversed with TP (Figure 3B). Together there was consistency between the reporter gene studies and western blot with regards to PTCH1 being a target of miR-9.

Next, we asked if miR-9 decreased PTCH1 in the TMZ resistant GBM cells. We stably transfected U87 and T98G cells with pPTCH-UTR-JM or control pPTCH-UTRΔ-JM (no miR-9-binding region). The transfectants were transiently transfected with anti-miR-9 or non-targeting oligos and then treated with 200 μM TMZ. After 72 h, the viable cells were studied for luciferase. Transfection of anti-miR-9 caused a significant (*p* < 0.05) increase in luciferase in the pPTCH-UTR-JM transfectants as compared to controls (Figure 3C, bars 2 and 3). MiR-9 was blocked from interacting with the 3′ UTR of PTCH by transfecting with pPTCH-UTRΔ-JM (miR-9 null site). The resulted in TMZ treatment luciferase activity that was similar to the anti-miR-9 (Figure 3C, bars 3 and 4). Together, the results indicated that miR-9 can suppress the translation of PTCH1 in TMZ resistant GBM cells. This occurred when miR-9 interacted with the 3′ UTR of PTCH1.

### MiR-9-PTCH1-SHH pathway

A decrease in PTCH1 would activate the SHH pathway. If so, this can be indicated by downstream Gli 1 (Figure 3F). We therefore performed cause-effect studies by ectopically expressing miR-9 in U87 and T98G cells and then determined if this reduced PTCH1 and increased Gli 1. GBM cells were stably transfected with pCMV-miR-9 and then studied for PTCH1 and Gli 1 by real time PCR and western blot. Ectopic expression of miR-9 and not the empty vector decreased PTCH1 and increased Gli 1 (Figures 3D–3G). This indicated that miR-9 can activate the SHH pathway, through a decrease in PTCH1.

### MiR-9 induced the expressions of drug transporters

Thus far, the studies supported a role for miR-9 in TMZ resistance of GBM cells. We asked if miR-9 is associated with increases in the drug transporters. U87 and T98G cells, stably expressing miR-9 or transfected with vector alone, were studied by real-time PCR and flow cytometry for the drug transporters, MDR1 and ABCG2. We previously reported in TMZ-induced MDR1 in GBM cells [26]. The results showed increases in the mRNA and protein for MDR1 and ABCG2 (Figures 3H–3K). These findings are consistent with other studies, which showed similar increases in the drug transporters by SHH signaling [31]. In summary, there were increases in membrane-bound MDR1 and ABCG2 in the miR-9 expressing GBM cells. These findings are consistent with miR-9 as an activator of a similar pathway (Figure 3F).

### Increased miR-9 and SHH signaling in primary GBM cells

We studied the significance of the findings with GBM cell lines by repeating key experiments with early passaged GBM cells from a patient who was never treated (BT145) and the compared with cells from a patient with recurrent (BT164) GBM. These two cell lines allowed us to compare a TMZ-resistant GBM cell line (BT164) with one that was never treated with TMZ (BT145).

**Figure 4:**
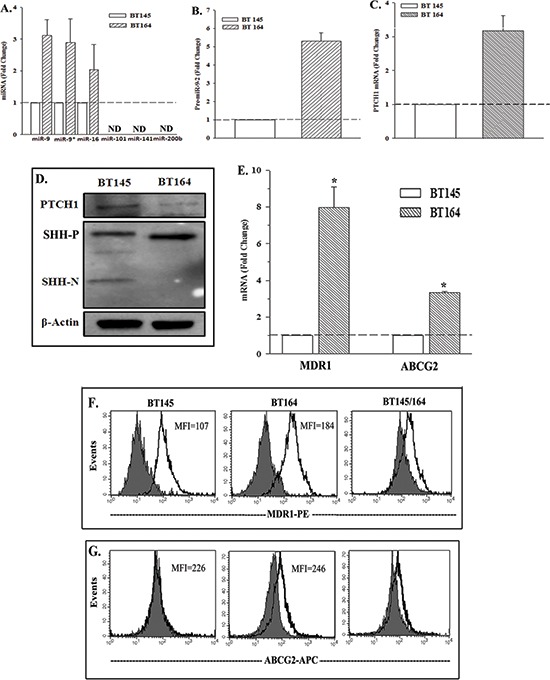
Expressions of PTCH1, miR-9-2 and drug transporters in naïve and recurrent GBM cells from patients **(A)** Real-time PCR was performed for the miRNA, predicted for PTCH1 (Figure 2A). The results are presented as the mean fold change ±SD, *n* = 4. **(B)** Real time PCR for miR-9-2 was performed in four independent studies using RNA from BT145 and BT164 cells. The results of BT145 were normalized to 1 and then used to calculate the fold change for the values of BT164, mean±SD. **(C)** Real time PCR were performed for PTCH1 as for ‘B’ and the results are similarly presented. **(D)** Whole cell extracts from BT145 and BT164 were studied by western blot for PTCH1, SHH-P (precursor), SHH-N (mature) and β-actin. **(E)** Real time PCR was performed for MDR1 and ABCG2 mRNA with total RNA from BT145 and BT164. **(F & G)** Flow cytometry was performed for membrane P-glycoprotein (*MDR1*) (F) and ABCG2 (G). The right panels show the overlay between the two patients. *p < 0.05 vs. BT145.

Real-time PCR showed increases in miR-9 and miR-16 in the recurrent BT164 cells (Figure 4A). MiR-9*, which is complementary to miR-9, was also upregulated, indicating that the increase in miR-9 was due to transcription and not stability. The increase in miR-9-2 was similar to the data from the two cell lines, U87 and T98G (Figure 2C). Real-time PCR also showed an increase in miR-9-2 in the recurrent BT164 cells (Figure 4B). We did not detect miR-9-1 or miR-9-3.

*PTCH1* expression in BT164 and BT145 was studied by real time PCR and western blot. The recurrent BT164 cells showed an increase in PTCH1 mRNA with reduced PTCH1 protein (Figures 4C, 4D). This disparity between PTCH1 mRNA and its protein was similar to the findings for TMZ resistant U87 and T98G cells (Figures 1A, 1B). Functional SHH (SHH-N) expression was not detected in BT164 cells (Figure 4D), indicating that the decrease in PTCH1 protein cannot be explained by receptor-mediated endocytosis and subsequent degradation of SHH. This observation was similarly detected for U87 and T98G cell lines (Figure 1).

### Increases in ABC transporters in the recurrent GBM tumor

TMZ resistant U87 and T98G showed increases in functional ABC drug transporters, ABCG2 and ABCB1 (MDR1) (manuscript submitted). In this study, ectopic expression of miR-9 in U87 and T98G resulted in increases in the same drug transporters (Figure 3). In this set of experiments we compared the naïve and recurrent BT145 and BT164 cell lines for the drug transporters. The results of real-time PCR were presented as fold change of BT164/BT145. There were 8 folds more for MDR1 and 4 folds for ABCG2 (Figure 4E). We next asked if the gene products of *MDR1* and *ABCG2*, P-glycoprotein and ABCG2, were transported to the cell surface. Flow cytometry indicated 40% greater MDR1 in BT164 as compared to BT145 (Figure 4F). The difference for ABCG2 expression was less, showing an increase in 10% (Figure 4G).

### Cell death of GBM cells by inhibitors of SHH signaling and P-gp

We investigated if the experimental findings can be translated to target GBM cells. The recently FDA-approved SHH inhibitor, GDC-0449 (Vismodegib (Vis), Erivedge^®^), has been shown to be effective in targeting SMO [32]. GDC-0449 inhibited SMO at <3 nM whereas at higher concentrations (3 μM), GDC-0449 inhibited both SMO and P-gp (ABCB1) [33]. To this end, we used different concentrations of GDC-0449 to inhibit SHH signaling and P-gp and studied how this treatment synergized with TMZ.

**Figure 5:**
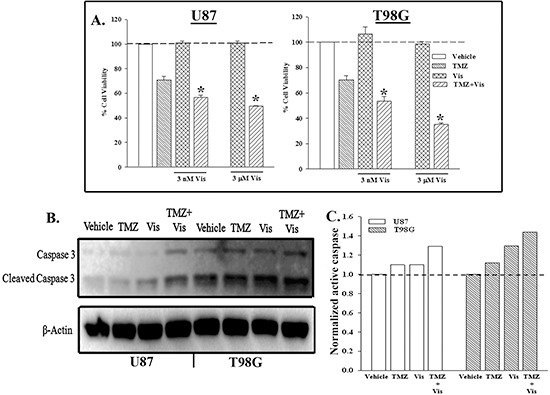
Combined treatment to inhibit SHH and P-gp enhanced cell death with TMZ treatment **(A)** U87 and T98G were treated with 3 nM, 3 μM GDC-0449 and/or 200 μM TMZ. After 72 h, cell viability was accessed by Cell Titer Blue. The percent cell viability is presented as mean±SD, *n* = 4. **(B)** Whole cell extracts from U87 and T98G, treated with 3 μM GDC-0449 and/or 200 μM TMZ. After 72 h, the cells were studied for caspase 3. **(C)** The band densities for active caspase 3 in ‘B’ were normalized with the bands for β-actin.

U87 and T98G cells were treated with 3 nM or 3 μM GDC-0449 (Vis) and/or 200 μM TMZ for 72 h. Cell viability showed significant decrease in viable cells as compared to TMZ or Vis alone (Figure 5A). We performed similar treatment as for Figure 5A, except with 3 μM GDC-0449 (Vis) to blunt both SMO and P-gp. Whole cell extracts were studied for active caspase 3 by western blot. The results showed 30% increase in cleaved caspase 3 (active) when the cells were treated with Vis and TMZ GDC-0449 (Vis) (Figures 5B, 5C).

## DISCUSSION

This study reported on a decrease in PTCH1 in GBM cells exposed to TMZ (Figure 1). This decrease was consistent with low-passage cell line from a patient with recurrent GBM (Figure 4). The decrease in PTCH1 was due to an increase in miR-9, resulting in TMZ resistance with concomitant increase in the drug transporter genes (Figure 4). As expected, the decrease in PTCH1 resulted in an increase in SHH signaling with increased in Gli, indicative of Smo activation. We therefore took advantage of this finding and use a pharmacological agent that is FDA approved in combination with TMZ to target SMO. The drug also targets the ABCG transporter at a higher concentration. Targeting both the SMO and drug transporter with TMZ led to significant cell death of the GBM cells (Figure 5). This latter finding was highly significant because the resistance of GBM to chemotherapeutic agents is a major factor in treatment failure [24].

The reactivation of SHH signaling in response to chemotherapy has been shown to play a major role in tumor refractoriness [24]. Although we also showed a similar finding, the pathway leading to SHH signaling was novel. We showed that resistance involved the post-transcriptional regulation of the PTCH1 receptor by miRNA-9. We also identified the miR-9-2 as the type among the three forms of this miR, which are derived at distinct chromosomes. The identified type (miR-9-2) has been shown to be involved in neural development.

We first showed a molecular dichotomy in which *PTCH1* mRNA is upregulated in response to TMZ treatment yet, PTCH1 protein did not increase when compared to untreated GBM cells (Figure 1). The expression of the ligand, SHH, was not involved in the resistance to TMZ (Figure 1). This indicated that a ligand-independent mechanism in PTCH1 regulation, rather than receptor-mediated endocytosis.

MiRNAs have been shown to have major roles in the post-transcriptional regulation of genes involved in cell death and developmental processes such as cell differentiation. In this report, we globally knockdown miRNAs by targeting *Dicer1* and showed an increase in the death of TMZ-treated GBM cells (Figure 1). These studies supported a role for miRNA in TMZ-resistance. Bioinformatic analyses of the 3′ UTR of *PTCH1* identified five potential miRNAs in the regulation of PTCH1. Among the miRNAs identified, only miR-9 was shown to be involved in TMZ resistance, specifically the *miR-9-2* gene (Figure 2). Using a cell line (CCL64) that was PTCH^+^/mir–9^−^ we demonstrated that endogenous *PTCH1* expression was suppressed by ectopic miR-9 (Figure 3). Furthermore, reporter gene studies with the PTCH1 3′ UTR and studies with target protector (TP) showed a direct regulation by miR-9 in the translation of PTCH1, through interaction with the 3′ UTR.

The significance identified with studies using GBM cell lines was supported by selected studies using miRNA data from The Cancer Genome Atlas (TCGA) (Figure 2E). The data showed increases in miR-9 expression, indicating that this miRNA must be important in GBM. In line with the observation in the TCGA, constitutive/ectopic expression of miR-9 in GBM cells confirmed that it can negatively regulate PTCH1 translation, resulting in the activation of SHH signaling (Figure 4). The central role of miR-9 in the resistance of GBM cells to TMZ led us to investigate the accompanied indicator of resistance. Indeed, we identified miR-9 involvement in the upregulation of the ATP-binding Cassette transporters, *MDR1* and *ABCG2* (Figure 4). At least for *MDR1*, this was functional because we used an antibody that detected the functional form of its protein, P-gp.

The significance of the finding with U87 and T98G cell lines was supported with patient-derived cell lines from naïve and recurrent (drug resistant) GBM. Unlike U87 and T98G, the cell lines from patients (BT145 and BT164) were subjected to limited passages. Comparison of these two cell lines showed the resistant line expressed miR-9-2 and decreased PTCH1 (Figure 4). Similar to U87 and T98G, the low-passaged cell lines also expressed *MDR1* and *ABCG2* (Figure 4). *MDR1* and *ABCG2* expressions have been shown to be downstream targets of SHH signaling. In this study, we showed that TMZ resistance is at least partly caused by the upregulation of miR-9, which in turn, regulated SHH signaling. The latter occurred by a decrease in its inhibitor, PTCH1. Together, this led to the upregulation of the drug efflux pumps.

Overall, we identified a number of potential targets to overcome GBM chemoresistance. Among these targets are miR-9, Dicer, SHH signaling, PTCH1, MDR1 and ABCG2. The FDA-approved Vismodegib (GDC-0449) with indications for a number of human malignancies, has been shown to inhibit SHH signaling and also, MDR1 and ABCG2. In this report, we identified GDC-0449 as a potentially important therapeutic agent when combined with TMZ to induced GBM cell death (Figure 5). We treated the GBM cells with TMZ and the concentration of GDC-0449 that inhibited both SHH and the drug transporters and showed a significant increase in cell death and caspase activation (Figure 5). The outcome with the combined treatment was significantly more efficient as compared to monotherapy with either drug.

In summary, we have identified a novel ligand-independent mechanism for the activation of SHH signaling, leading to drug efflux-mediated chemoresistance of GBM cells. We were able to support the findings with immortalized cell lines from GBM patients, with limited passages and with miRNA arrays from the tumor atlas. We translated the molecular findings with the FDA-approved Vismodegib to overcome the chemoresistance of GBM, showed enhanced cell death. These studies suggested that the addition of drug efflux inhibitors and SHH inhibitors may enhance GBM cell death and prevent the chemoresistance properties uniformly seen in this human malignancy. Yet, the experimental evidence indicated that miRNAs and its anti-miRs could be used in molecular therapy to target GBM. Currently, there are methods to develop how stem cells can be used as a cellular vehicle to deliver RNA [34]. The transport of miRNAs from stem cells to cancer cells has been shown to be mediated through gap junctional communication as well as through the secretion of exosomes [35]. However, as RNA therapeutics develop, targeting miR-9 should be the most efficient to overcome its induced pathway in the chemoresistance of GBM.

## MATERIALS AND METHODS

### Cells

All cell lines used in the study ensures authentication, as discussed in this section. U87 and T98G, WHO-grade IV glioblastoma cells, HEK293, and CCL64 lung epithelial cells were purchased from American Type Culture Collection (ATCC; Manassas, VA). The cells were expanded as per manufacturer’s instructions and then cryopreserved as passage 1 cells. The experiments were performed with thawed cells up to passage 5.

BT145 (primary GBM) and BT164 (recurrent GBM) glioma cell lines were derived from surgical resection material acquired from patients undergoing surgery at the Brigham and Women’s Hospital on an Institutional Review Board approved protocol as part of the DF/BWCC Living Tissue Bank. Briefly, tumor resection samples were mechanically dissociated and tumorspheres were established and propagated in Human NeuroCult NS-A Basal media (StemCell Technologies) supplemented with EGF, FGFβ and heparin sulfate.

### Reagents and antibodies

All tissue culture media were purchased Life Technologies (Grand Island, NY), fetal calf serum (FCS) from Hyclone Laboratories (Logan, UT), TMZ from Sigma Aldrich (St. Louis, MO) and GDC-0449 (Vismodegib, Erivedge^®^), from LC Laboratories (Woburn, MA).

Rabbit anti-human PTCH1 was custom-ordered from Novoprotein (Short Hills, NJ). Rabbit anti-SHH, -Dicer, -Gli1, -Caspase 3; murine anti-β-actin mAb, HRP-anti-rabbit and HRP-anti-mouse IgG were purchased from Cell Signaling (Danvers, MA). Murine anti-human P-gp (UIC2 clone)-PE and anti-human ABCG2-APC were purchased from Biolegend (San Diego, CA).

### siRNA

*SHH* and *Dicer1* targeting siRNAs were purchased from Thermo Scientific (Waltham, MA); precursor miR-9 (pre-miR-9) and antisense miRNA-9 (anti-miR-9) were purchased from Life Technologies. All siRNA, pre-miRs and anti-miRs were transfected with Lipofectamine RNAiMax (Life Technologies).

### Real-time RT-PCR

RNA was extracted with Trizol reagent (Invitrogen) and then reverse transcribed with the High Capacity cDNA Reverse Transcription Kit (Applied Biosystems). PCR was performed with 200 ng of cDNA using Taqman MicroRNA Assays with specific miRNA primers (Table S1). The amplicons were normalized with RNU6B. The primers to recognize the microRNA-9 primary molecules were previously reported and are presented in Table S2 [29].

Real-time PCR was performed on 7300 PCR System (Applied Biosystems) as follows: an initial incubation of 50°C for 2 min followed by 95°C for 10 min. After this, the cycling conditions were as follows: 95°C for 15 s and 60°C for 60 s, 40 cycles. Table S1 shows the primer sequences, which were obtained from Sigma. The relative expression was calculated using the 2(−Delta Delta C(T)), as previously described [35].

### Western blot

GBM cells were untreated and treated with vehicle or 200 μM TMZ. At different times, whole cell extracts were isolated with M-PER Mammalian Protein Extraction Reagent (Thermo Scientific). The extracts (3–7 μg) were analyzed by western blots with 12% SDS-PAGE (Bio-Rad, Hercules, CA), as described [36]. Proteins were transferred onto PVDF membranes (Perkin Elmer, Boston, MA). The membranes were incubated overnight with primary antibodies at final dilutions of 1/500-1000. Primary antibodies were detected during a 2-h incubation with HRP-conjugated IgG at 1/2000 final dilution. HRP activity was detected by chemiluminescence using SuperSignal West Femto Maximum Sensitivity Substrate (Thermo Scientific). Membranes were stripped with Restore Stripping Buffer (Thermo Scientific) for reprobing with other antibodies.

### Flow cytometry

The expressions of P-gp and ABCG2 on cell membranes were studied by flow cytometry with murine mAb conjugated to PE (P-gp) or to APC (ABCG2). P-gp expression was analyzed using the UIC2 clone and ABCG2 expression was analyzed using the 5D3 clone. The cells were immediately analyzed on a FACS Calibur II (BD Biosciences, San Jose, California) and the data were analyzed with the FlowJo software (BD Biosciences).

### Vectors and transfectants

The reporter gene vector containing the PTCH1 3′ UTR was custom ordered from Origene Technologies (Rockville, MD). The sequence was ligated in pMirTarget, upstream of the RFP and luciferase genes and designated, pPTCH-UTR-JM. A mutant form of pPTCH-UTR-JM was generated by deleting the miR-9 binding site within the 3′ UTR, designated pPTCH-UTRΔ-JM. The deletion was achieved with the Expand Long Template PCR System (Roche, Indianapolis, IN) and the following primers: Forward, 5′-ACG CGT AAC CTT TGG GGG GTG G-3′ and Reverse, 5′-CGC CGG CGC CCT CAT TAT TAG GCA TC-3′. The primers were synethesized with the sequences for *Sgf1* and *Mlu1* (New England Biolabs, Ipswich, MA). Direct insertion of the amplified fragment was placed into pMirTarget using T4 DNA ligase (Clontech, Mountain View, CA). The deletion of the miR-9 site was confirmed by DNA sequencing at Genewiz (South Plainfield, NJ).

pCMV-miR co-expressing miR-9-2 and GFP, was purchased from Origene Technologies and then stably transfected in U87 and T98G cells with Effectene (Qiagen, Valencia, CA). The stable transfectants were selected with 200 μg/mL of G148 (Invitrogen/Life Technologies).

### Transfection and reporter gene assay

CCL64, U87 and T98G cells were stably expressed with pPTCH-UTR-JM. The vector was transfected with Effectene Transfection Reagent (Qiagen, Valencia, CA) and then selected with 200 μg/mL of G418. The CCL64 transfectants were transiently transfected with pre-miR-9 and/or anti-miR-9 using Lipofectamine RNAiMAX. U87 and T98G cells were instead exposed to 200 μM TMZ for 72 h. Protein lysates were obtained with M-PER Mammalian Protein Extraction Reagent and then quantitated for total protein using the Bio-Rad Protein Kit (Hercules, CA). Luciferase activity was normalized to total protein and calculated as relative light units (RLU). The relative expression was calculated by normalizing to control (untreated) RLU.

Site specificity for miR-9 in luciferase activity was verified by repeating the transient transfections, except with the addition of target protection (TP) assay (Qiagen). The oligos were custom-made to specifically block mir-9 binding to the PTCH1 3′ UTR. The TP and negative control oligos were transfected with Lipofectamine RNAiMax.

### MiRNA analysis of the 3′ UTR of *PTCH1*

The 3′ UTR of *PTCH1* was identified from the NCBI database (NM_000264) beginning after the TGA terminal codon of the transcript, from +3425 followed by the poly-A tail domain. The entire sequence was analyzed for potential interacting miRNAs using a combination of programs: Target Scan^®^, mirBase^®^, and miRDB^®^. The miRNAs were selected based on sequence complementarity and species conservation.

### Analyses of the miRNA database from the cancer genome atlas (TCGA)

Level 3 expression data for miRNAs were obtained from TCGA for GBM (https://tcga-data.nci.nih.gov/tcga/). The miRNA showing +/−0.5 fold changes were considered significant.

### Cell viability assay

Cell viability was determined by treating GBM cells with 200 μM TMZ for 72 h in 96-well tissue culture plates. The cells were seeded at 1.5 × 10^4^/well in 150 μL of media. Cell viability was assessed with the Cell Titer Blue Cell kit (Promega, Madison, WI).

### Statistical analyses

Data were analyzed using the paired *t*-test for two comparable groups (control vs. experimental). A  *p* value < 0.05 was considered significant.

## SUPPLEMENTARY FIGURES AND TABLES


