# Supplementary material for: Temozolomide resistance in glioblastoma occurs by miRNA-9-targeted PTCH1, independent of sonic hedgehog level
Source: Oncotarget. 2015 Feb 6;6(2):1190–201. doi: 10.18632/oncotarget.2778 (PMC4359226; doi:10.18632/oncotarget.2778)
Supplement: Supplementary file 1 [file oncotarget-06-1190-s001.pdf]

SUPPLEMENTARY FIGURES AND TABLES

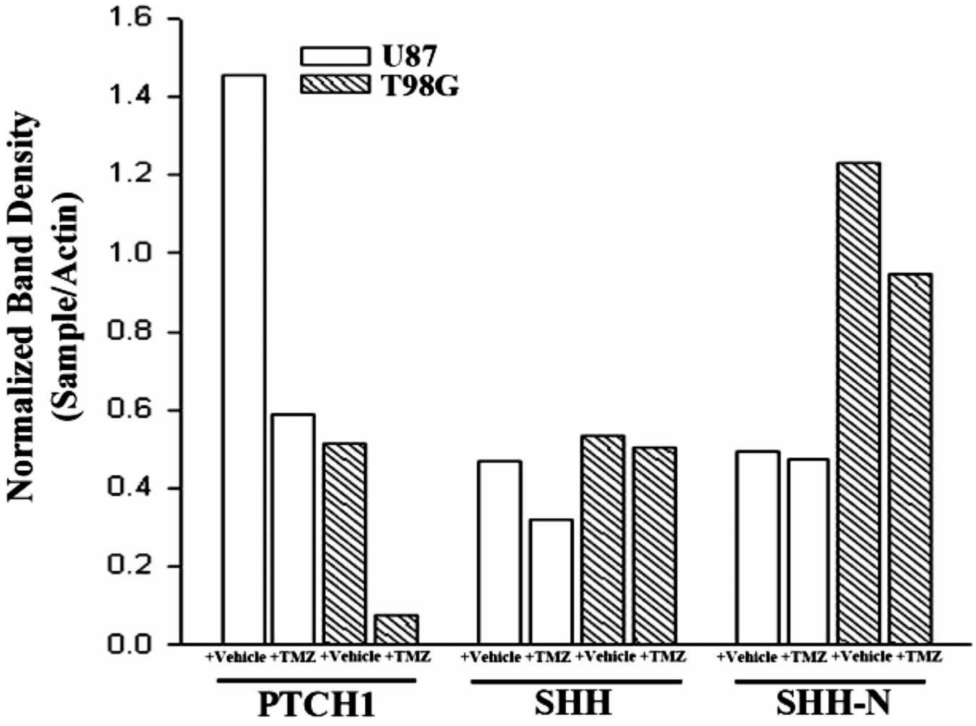

Supplementary Figure 1: Band densities for Fig. 1B, normalized with bands for β-actin.

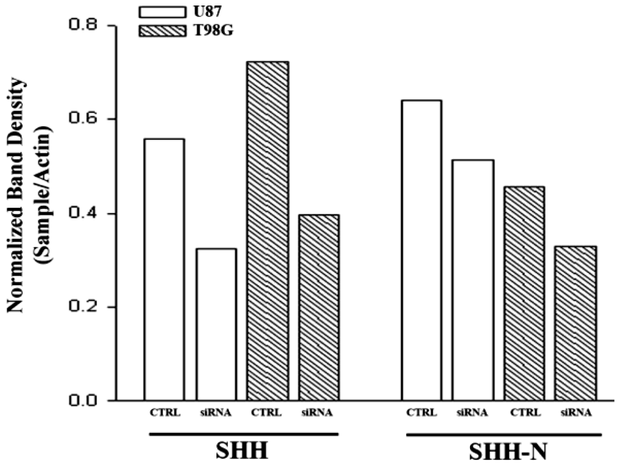

Supplementary Figure 2: Band densities for Fig. 1C, normalized with bands for β-actin.

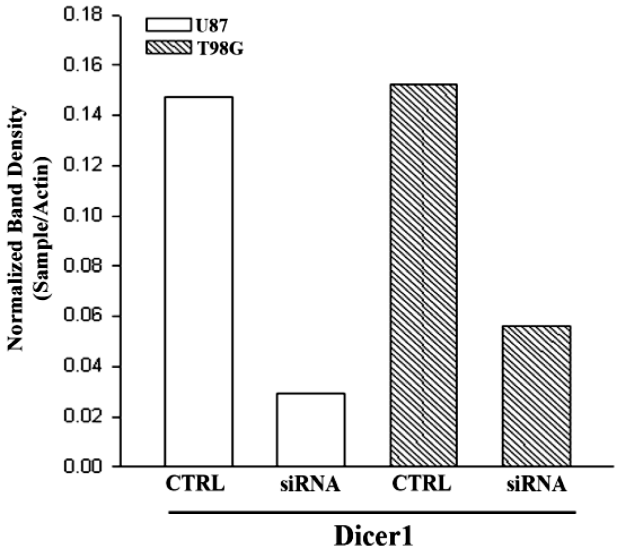

Supplementary Figure 3: Band densities for Fig. 1E, normalized with bands for β-actin.

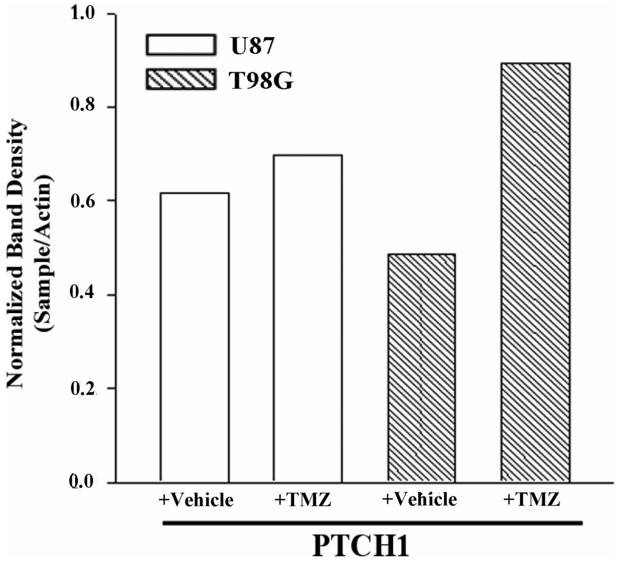

Supplementary Figure 4: Band densities for Fig. 1F, normalized with bands for β-actin.

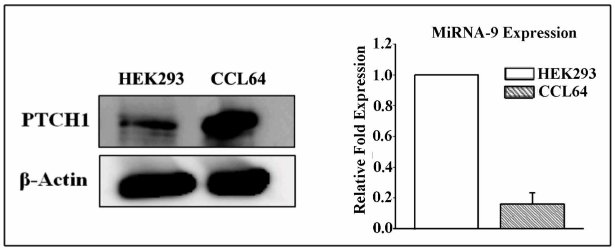

Supplementary Figure 5: CCL64 cells were studied for PTCH1 by western blot (left). Real time (right). HEK293 cells served as a positive control for PTCH1.

Supplementary Table S1: Taqman® primer sequences

Targets	Taqman Sequence
**miR-9**	5′-UCU UUG GUU AUC UAG CUG UAU GA-3′
**miR-101**	5′-UAC AGU ACU GUG AUA ACU GAA-3′
**miR-15/16a**	5′-UAG CAG CAC AUA AUG GUU UGU G-3
**miR-200a**	5′-UAA CAC UGU CUG GUA ACG AUG U-3′
**RNU6B**	5′-CGC AAG GAT GAC ACG CAA ATT CGT GAA GCG TTC CAT ATT TTT-3′

Supplementary Table S2: Primers used in real-time PCR

Transcript	Forward Primer (5′…3′)	Reverse Primer (5′…3′)	Region
**PTCH1**	GTT GCA GCG TTA AAG GAA	CCA GCG GCT ACT TAC TCA	1516/1667
**Gli1**	CCA ATG AGA AGC CGT ATG	TGG GCT CCA CTG TAG AAA	1148/1304
**MDR1**	TCA GGT GGC TCT GGA TAA	CTG CTG TCT GCA TTG TGA	2217/2370
**ABCG2**	GTG GCC TTG GCT TGT ATG AT	GAT GGC AAG GGA ACA GAA AA	2229/2389
**β-Actin**	TGC CCT GAG GCA CTC TTC	GTG CCA CCA GGG CAG TGA TCT	870/1031
